# Follow-Up Diffusion-Weighted Image Reveals Delayed Appearance of Ischemic Lesions in Suspected Transient Ischemic Attack

**DOI:** 10.7759/cureus.47405

**Published:** 2023-10-20

**Authors:** Hiroyuki Kida, Kenichiro Sakai, Takeo Sato, Ryoji Nakada, Tomomichi Kitagawa, Hiroki Takatsu, Teppei Komatsu, Kenichi Sakuta, Hidetaka Mitsumura, Yasuyuki Iguchi

**Affiliations:** 1 Department of Neurology, Jikei University School of Medicine, Tokyo, JPN

**Keywords:** follow-up mri, delayed ischemic lesion, transient ischemic attacks, ais (acute ischemic stroke), diffusion weighted imaging (dwi)

## Abstract

Introduction: In patients suspected of transient ischemic attack (TIA), it is not uncommon to find no lesion on the diffusion-weighted image (DWI) on admission but a delayed appearance on the follow-up DWI.

Methods: Enrolled patients met the following criteria: (1) MRI performed within 24 hours of onset and seven days after admission; (2) National Institutes of Health Stroke Scale (NIHSS) score ≦4 on admission; (3) pre-stroke modified Rankin scale (mRS) score of 0-1. Patients were divided as follows: no lesion on the first DWI and a new lesion on the second DWI (delayed-specified ischemic stroke; DSIS); and no lesion on either the first or second DWI (well-screened TIA; WSTIA). We compared both groups regarding the clinical background and the outcome at three months.

Results: We identified 144 cases (male 70%; median age 64 years; DSIS, n=34) between October 2012 and March 2019. DSIS was older (71 vs. 60 years, *p*=0.006) and had a higher NIHSS score on admission (1 vs. 0, *p*=0.041), a higher rate of large vessel occlusion (LVO) (17% vs. 2%, *p*=0.008),* *and symptom duration over one hour (82% vs. 64%, *p*=0.041). A favorable outcome mRS score of 0-1 at three months was less frequent in DSIS (85% vs. 96%, *p*=0.004). Age/10 (OR 1.62, 95%CI 1.17-2.24; *p*=0.004) and LVO (OR 10.84, 95%CI 1.87-63.06; *p*=0.008) were independent factors for DSIS.

Conclusions: In suspected TIA with age or LVO but no lesion in the initial DWI, the second DWI should be considered to identify the delayed appearance of an ischemic stroke.

## Introduction

Transient ischemic attack (TIA) is a significant risk for acute ischemic stroke (AIS) [1]. Several guidelines recommend the rapid screening of etiology and therapeutic intervention for TIA patients [2,3].

TIA has been defined as “a neurologic deficit lasting less than 24 hours that is attributed to focal cerebral or retinal ischemia” (time-based definition) [4]. Magnetic resonance imaging (MRI) can identify acute ischemic lesions, and a previous study reported that in 34% of patients with TIA, according to the time-based definition, positive ischemic lesions were apparent on diffusion-weighted imaging (DWI) [5]. As the outcomes of DWI-positive and time-based TIA resemble those of ischemic stroke, the International Classification of Diseases (ICD)-11 definition of TIA additionally requires “without acute infarction in the clinically relevant area of the brain” (a tissue-based definition) [6]. Consequently, the diagnosis of TIA can be converted to that of ischemic stroke depending on the results of neuroimaging, even if the neurological deficit disappears within 24 hours, but generally within one hour [7]. In patients with an acute neurological deficit, mild (or even resolved) symptoms, and no ischemic lesion on DWI, the first diagnosis might be TIA. However, it is not uncommon for an ischemic lesion to appear on a second or later DWI in TIA-suspected patients. In such cases, the final diagnosis becomes ischemic stroke (delayed-specified ischemic stroke; DSIS). The frequency, clinical background, and outcome of DSIS in TIA-suspected patients are insufficiently understood.

The aim of the study is to identify differences in clinical background and outcome between DSIS and tissue-based TIA in patients presenting with suspected TIA to predict whether suspected TIA would be DSIS or TIA from the clinical information on the admission.

## Materials and methods

Subjects

**Figure 1:**
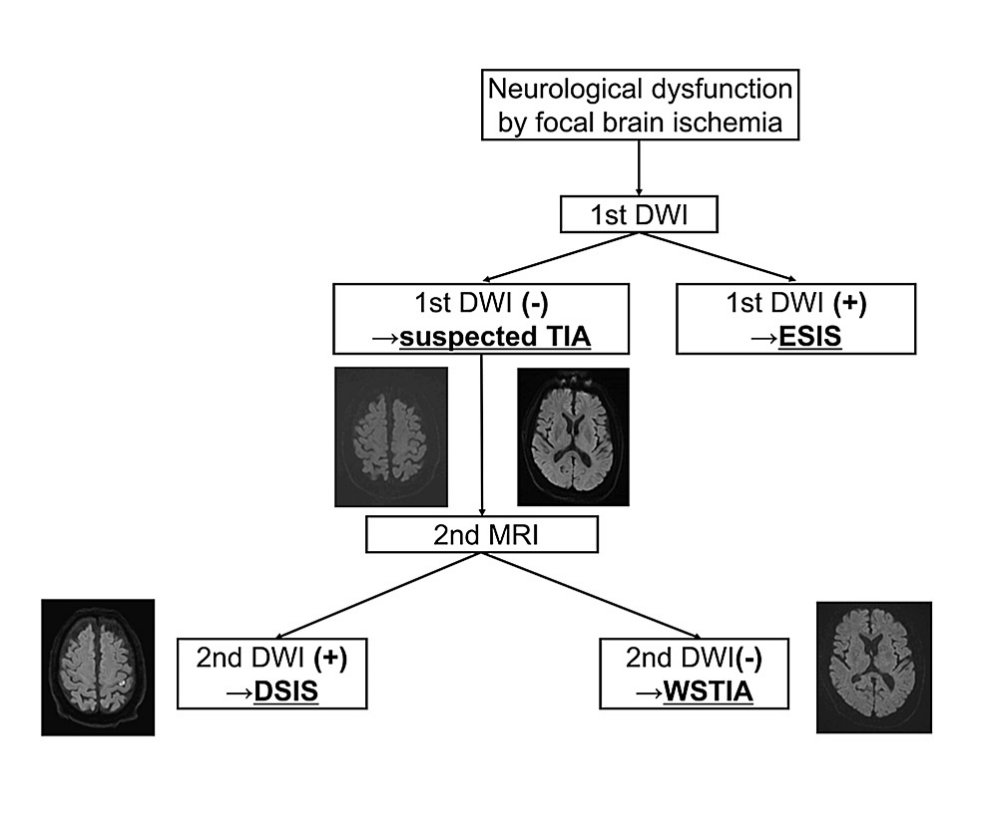
Diagnoses based on the diffusion-weighted imaging findings. The ICD-11 definition of TIA is a tissue-based classification with the following criteria: (1) without acute infarction in the area clinically relevant to the focal neurological dysfunction, and (2) complete resolution of symptoms within 24 hours. ESIS, early-specified ischemic stroke; DSIS; delayed-specified ischemic stroke; DWI, diffusion-weighted imaging; TIA, transient ischemic attack; WSTIA, well-screened transient ischemic attack.

Between October 2012 and March 2019, patients with acute ischemic stroke (AIS) and TIA were obtained retrospectively from the database of the Jikei University School of Medicine Stroke Registry (Jikei Stroke Registry), a prospective database of patients with acute stroke who were admitted to Jikei University Hospital, a tertiary medical center located in the metropolitan area of Tokyo, Japan. The inclusion criteria were as follows: (1) DWI performed both within 24 hours from symptom onset and within seven days after admission; (2) National Institutes of Health Stroke Scale (NIHSS) score on admission less than 4; (3) pre-stroke modified Rankin scale (mRS) score of 0-1 [8,9]. Patients with acute neurologic dysfunction on admission underwent DWI, and those with positive findings were excluded as having an early-specified ischemic stroke (ESIS) on admission. Patients with negative findings on the first DWI were termed suspected TIA. In our protocol for TIA, or ischemic stroke, we conduct a follow-up MRI within seven days after admission. Based on the results of the second DWI, patients with an ischemic lesion on this second DWI were classified as having a DSIS, and those with no lesion on the second DWI had a well-screened TIA (WSTIA) (shown in Figure 1). All imaging and stroke/TIA diagnoses were reviewed by at least two vascular neurologists.

Clinical background

Baseline data included sex, age, NIHSS score on admission, systolic and diastolic blood pressure on admission, past history of cardiovascular disease risk factors (hypertension, diabetes mellitus, dyslipidemia, and atrial fibrillation), past history of any stroke/TIA, time course of stroke, intravenous thrombolysis, mechanical thrombectomy, antithrombotic drug use (antiplatelet therapy or anticoagulative therapy), past histories of risk factors for stroke, and pre-stroke mRS score were obtained by physicians during medical interviews with the participant or the substitute guardians.

Comorbidities and risk factors for stroke were defined as: (1) hypertension, use of antihypertensive agents before admission or at discharge; (2) diabetes mellitus, use of oral hypoglycemic agents or insulin, diet therapy, and random blood glucose level ≥200 mg/dl or fasting plasma glucose level ≥126 mg/dl with glycosylated hemoglobin level (National Glycohemoglobin Standardization Program) ≥6.5%; (3) dyslipidemia, use of cholesterol-lowering drugs, or serum LDL-cholesterol ≥140 mg/dl; (4) smoking, any lifetime use of cigarettes; and (5) body mass index.

The source of embolism was defined as a high- or medium-risk cardioembolism subtype of the Trial of Org 10172 in the Acute Stroke Treatment (TOAST) classification, as extracranial or intracranial atherosclerosis causing >50% luminal stenosis in the arteries supplying the area of ischemia, or as an aortic complicated lesion (>4 mm or ulcered plaque on the aortic arch) [10]. The embolic source was identified by the following mandatory examinations: carotid duplex ultrasound (CUS), Holter echocardiogram, computed tomography angiography (CTA), magnetic resonance angiography (MRA), transcranial Doppler, transthoracic echocardiogram, and/or transesophageal echocardiogram.

The time course of stroke included: (1) duration of symptoms; (2) interval from onset to first DWI; and (3) interval from onset to second DWI. The ABCD2 score comprised age (≥60 years, 1 point), blood pressure (systolic ≥140 mmHg or diastolic >90 mmHg, 1 point), clinical features (unilateral weakness, 2 points; isolated speech disturbance, 1 point), duration of symptoms (≥60 min, 2 points; 10-59 min, 1 point), and diabetes (present, 1 point) [11]. Large vessel occlusion (LVO) was defined as the occlusion of an intra- or extracranial artery such as the ICA, ACA (A1, A2), MCA (M1, M2), VA, BA, or PCA (P1, P2) [12]. All LVOs were considered clinically relevant to the neurological deficit and were confirmed by MRA, CUS, or CTA. As the cohort had mild symptoms on admission, neurological deterioration was defined as an increase of 2 or more NIHSS points [13,14]. The outcome at three months after onset was categorized as favorable for an mRS score of 0-1, and unfavorable for an mRS score of 2-6.

Neuroimaging

Three MRI systems were used in this study. MAGNETOM Avanto and MAGNETOM Symphony (Siemens, Erlangen, Germany) at 1.5 T with the following sequence parameters for DWI: repetition time (TR), 2700 ms; echo time (TE), 90 ms; section thickness, 5 mm; matrix, 128 × 128; and field of view (FOV), 21 cm. We also used a MAGNETOM Skyra system (Siemens, Forchheim, Germany) at 3.0 T with the following sequence parameters for DWI: TR, 5000 ms; TE, 65 ms; section thickness, 4 mm; matrix, 160 × 160; and FOV, 22 cm.

Statistical analysis

The clinical characteristics of patients were compared between DSIS and WSTIA using the chi-squared test, Fisher’s exact test, and the Mann-Whitney U-test. The factors able to be evaluated on admission and having a probability of <0.05 in univariable analysis were entered into a multivariable analysis to determine the adjusted odds ratios of factors associated with DSIS. Data were statistically analyzed using the commercially available software package SPSS version 22 (SPSS Japan, Inc., Tokyo, Japan). Differences were considered statistically significant at the level of p < 0.05.

Standard protocol approvals and registrations

This study conformed to the ethical principles established in the Declaration of Helsinki, and the study protocol was approved by the Institutional Review Board at Jikei University School of Medicine (No. 8813) in accordance with the Ethical Guidelines for Medical and Health Research Involving Human Subjects in Japan [15]. The board waived the need for patient consent.

## Results

**Figure 2:**
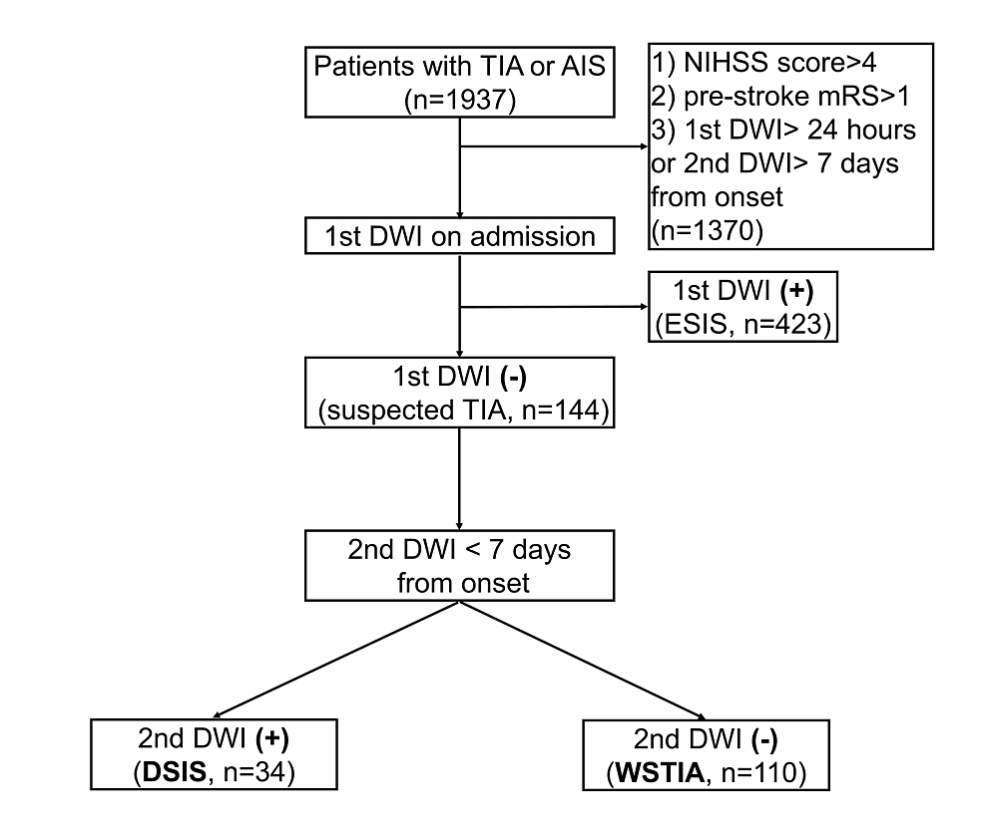
Patient classification. Patients with TIA and minor ischemic stroke (NIHSS score £4) with pre-stroke mRS score of 0–1 and no ischemic lesion on the first DWI were termed suspected TIA. Patients with suspected TIA were divided into DSIS and WSTIA according to the findings of the second DWI. AIS: acute ischemic stroke; ESIS: early-specified ischemic stroke; DSIS: delayed-specified ischemic stroke; DWI: diffusion-weighted imaging; mRS: modified Rankin scale; NIHSS: National Institutes of Health Stroke Scale; TIA: transient ischemic attack; WSTIA: well-screened transient ischemic attack.

Among the 1,937 patients admitted to our hospital with AIS and TIA, 1,793 patients were excluded, and a total of 144 suspected TIA patients were analyzed (male 70%, median age 64 years). Of these, 34/144 (24%) were DSIS and 110/144 (76%) were WSTIA (shown in Figure 2). The clinical characteristics of the two groups are listed in Table 1. Among the patient characteristics, age, NIHSS score on admission, identified source of embolism, and LVO were higher for DSIS than for WSTIA. There was no significant difference between the groups in terms of the frequency of vascular risk factors or atrial fibrillation.

**Table 1:** Patient characteristics. DSIS: delayed-surveyed ischemic stroke; DWI: diffusion-weighted image; mRS: modified Rankin scale; NIHSS: National Institutes of Health Stroke scale; WSTIA: well-surveyed transient ischemic attack.

	Total (n=144)	DSIS (n=34)	WSTIA (n=110)	p-value
Age, y, median (IQR)	64 (52–76)	71 (63–79)	60 (51–74)	0.006
Male, n, %	101 (70)	25 (74)	76 (69)	0.621
Past history				
Hypertension, n, %	91 (63)	23 (68)	68 (62)	0.538
Hyperlipidemia, n, %	77 (54)	19 (56)	58 (53)	0.747
Diabetes mellitus, n, %	25 (17)	5 (15)	20 (18)	0.640
Atrial fibrillation, n, %	15 (10)	6 (18)	9 (8)	0.114
Pre-stroke mRS score, median (IQR)	0 (0-0)	0 (0-0)	0 (0-0)	0.906
Identified source of embolism during admission	58 (40)	21 (62)	37 (34)	0.003
Symptom duration over 1 hour, n, %	98 (68)	28 (82)	70 (64)	0.041
Time from onset to first DWI, min, median (IQR)	144 (85–237)	148 (85–189)	144 (87–240)	0.821
Time from onset to second DWI, min, median (IQR)	1610 (1255-3017)	1613 (1176–3028)	1610 (1290–2984)	0.552
NIHSS score on admission, median (IQR)	0 (0–1)	1 (0–2)	0 (0–1)	0.041
Persisting symptoms on admission, n, %	65 (45)	20 (59)	45 (41)	0.067
ABCD^2^ score, median (IQR)	4 (3-5)	5 (3-5)	4 (4-5)	0.116
Neurological deterioration, n, %	6 (4)	5 (15)	1 (1)	0.003
Large vessel occlusion, n, %	7 (5)	5 (17)	2 (2)	0.008
Thrombolysis, n, %	8 (6)	2 (6)	6 (6)	0.924
Mechanical thrombectomy, n, %	2 (1)	1 (3)	1 (1)	0.376
Antithrombotic drug use, n, %	134 (94)	34 (100)	100 (92)	0.083

Regarding clinical time course, the duration of symptoms over one hour was higher for DSIS than for WSTIA. There was no significant difference between DSIS and WSTIA in terms of ABCD2 score (5 vs. 4, p=0.116) or treatment (thrombolysis, mechanical thrombectomy, and antithrombotic drug usage). Neurological deterioration occurred in six patients (DSIS in 5 and WSTIA in 1, 15% vs. 1%, p=0.003, Table 1).

Of the DSIS patients, neurological imaging revealed a single lesion in 27/34 patients (79%) and multiple lesions in 7/34 (21%). Twenty-two (65%) of DSIS patients had an ischemic lesion in the anterior circulation. Lesions were localized to the cortex (n=13), subcortex (n=17), cerebellum (n=2), and brainstem (n=10).

**Figure 3:**
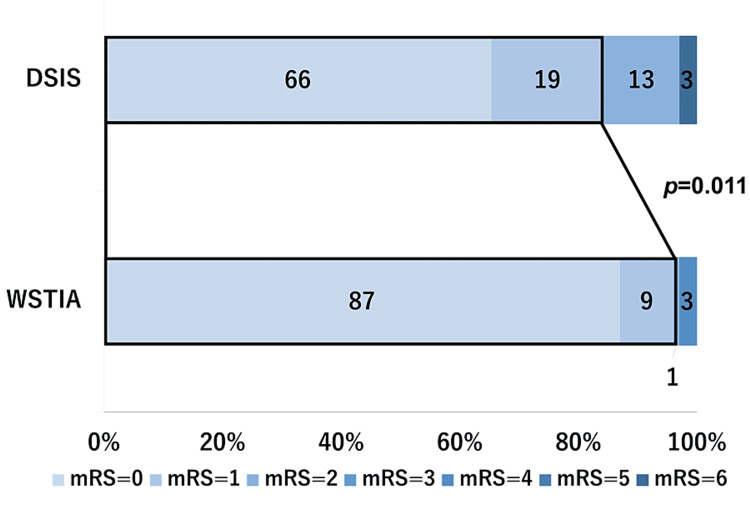
Outcomes at three months after onset. The prevalence of favorable outcome (mRS score 0–1) at three months after admission was significantly lower in DSIS patients than in WSTIA patients (85% vs. 96%, p=0.011). DSIS: delayed-specified ischemic stroke; mRS: modified Rankin scale; WSTIA: well-screened transient ischemic attack.

The prevalence of favorable outcomes at three months from onset was significantly lower for DSIS compared with WSTIA (85% vs. 96%, p=0.011, shown in Figure 3). The multivariable logistic regression analysis demonstrated age/10 (OR 1.62, 95%CI 1.17-2.24; p=0.004) and LVO (OR 10.84, 95%CI 1.87-63.06; p=0.008) as independently associated with DSIS (Table 2).

**Table 2:** Multivariable analysis for predicting delayed-surveyed ischemic stroke. CI: confidence interval; OR: odds ratio; NIHSS: National Institutes of Health Stroke scale.

	OR	95%CI	p value
Age/10	1.62	1.17–2.24	0.004
Symptoms duration > 1 hour	1.71	0.58–5.02	0.328
NIHSS score on admission	1.41	0.95–2.09	0.091
Large vessel occlusion	10.84	1.87–63.06	0.008

## Discussion

The present study revealed three major findings of interest: 24% of suspected TIA cases were finally categorized as DSIS, favorable outcomes were less prevalent in DSIS than in WSTIA, and age and LVO were independent factors associated with DSIS in suspected TIA patients.

In one-quarter of patients who had minor stroke-related symptoms and no lesions on the initial DWI, ischemic lesions were apparent on the second DWI. This finding is in agreement with a previous study that reported almost 18% for the same definition of DSIS; however, in contrast with the present study, they did not compare clinical backgrounds between DSIS and WSTIA, including the prevalence of LVO and neurological deterioration [16].

Our study showed that, compared with WSTIA patients, DSIS patients more frequently deteriorated with an unfavorable outcome, while the ABCD2 score was not significantly different between the two groups. It indicated that DSIS might be associated with unfavorable outcomes independently of traditional risk scores. A previous study reported that patients with DWI-positive ischemic lesions according to time-based TIA were more likely to relapse, and their outcomes tended to be worse compared with DWI-negative patients [17,18]. For time-based TIA, the prevalence of ischemic stroke within 90 days has been reported at 24%, some of which were thought to have the same pathophysiology as DSIS [19]. Thus, early MRI following, that is, detecting DSIS, may be useful as an important biomarker for predicting the outcome in patients considered to have TIA or a minor stroke.

Age was an independent factor associated with DSIS, perhaps for the reason that patients with stroke mimics such as seizures and functional disorders tend to be young [20]. WSTIA patients were younger than DSIS patients, which means that the WSTIA group could have included stroke mimics even though we took care to exclude these patients. In addition, the embolic source varied in DSIS patients but was most frequently a cortical ischemic lesion, which suggests embolic stroke. Taking into consideration the decreased cerebral blood flow in older patients, embolism occurs more frequently in this age group, and “wash-out” is impaired due to low cerebral blood flow [21,22]. Thus, these factors may increase the likelihood of delayed ischemic lesions in elderly TIA patients.

LVO was also an independent factor in DSIS, partly because of the greater number of sources of embolism in DSIS patients. Another possible explanation is that DSIS with LVO could indicate the appearance of a new lesion rather than the delayed appearance of the initial infarction, as LVO is associated with neurological deterioration [10]. In a clinical situation, some DSIS patients may be suitable candidates for urgent endovascular therapy. Thus, vascular imaging should not be spared even in suspected TIA, and careful follow-up DWI is important even when no ischemic lesion can be identified on the initial DWI.

As mentioned above, a diagnosis of TIA can be changed to DSIS if the second DWI is positive. Because embolism is common in DSIS, intensive detection of the embolic source by transesophageal echocardiography and extended cardiac monitoring can prevent a relapse of ischemic stroke. In addition, intensive therapeutic intervention such as dual antiplatelet therapy (DAPT) might decrease the recurrence of ischemic stroke. However, the optimal duration of DAPT for an individual patient is unknown, and the diagnosis of DSIS may be a biomarker for recommending long-term (for example, three months) DAPT. In TIA or minor stroke, DSIS can be a supportive biomarker to prolong DAPT. Thus, to improve outcomes following TIA, especially in elderly patients, identification of DSIS by follow-up DWI is considered.

Our study has several limitations. We were unable to completely exclude stroke mimics such as seizures or functional disorders. It was difficult to differentiate DSIS between the delayed appearance of initial ischemia and the occurrence of brand-new ischemia. The latter case should be classified as ESIS. A lesion smaller than the DWI thickness of 5 mm could not be detected by DWI. This is also a retrospective, single-facility, and small-number study.

## Conclusions

In conclusion, DSIS is not uncommon in the tissue-based definition of TIA. As the outcome of DSIS is unfavorable, a second DWI should be performed within seven days of the DWI obtained in suspected TIA patients at admission, especially in elderly patients and in those with LVO.
